# Lipid ligand binding and membrane interactions of a novel food-derived lipid transfer protein enhance basophil allergic responses

**DOI:** 10.1038/s41598-026-55182-9

**Published:** 2026-06-13

**Authors:** Uta Jappe, Jochen Behrends, Andra B. Schromm

**Affiliations:** 1 Division of Clinical and Molecular Allergology, Research Center Borstel, Leibniz Lung Center, German Center for Lung Research (DZL), Airway Research Center North (ARCN), Borstel, Germany; 2 Interdisciplinary Allergy Outpatient Clinic, Department of Pneumology, UKSH, University of Luebeck, Luebeck, Germany; 3 Science and Technology Unit Fluorescence Cytometry, Research Center Borstel, Leibniz Lung Center, Borstel, Germany; 4 Division of Immunobiophysics, Research Center Borstel, Leibniz Lung Center, Borstel, Germany

**Keywords:** Anaphylaxis, Allergen-lipid interaction, Lipophilic allergens, Lipid-transfer-protein, Basophil activation test (BAT), LTP-membrane interaction, Förster-resonance-energy-transfer assay (FRET), Biochemistry, Biotechnology, Drug discovery, Immunology

## Abstract

Non-specific (ns) lipid transfer proteins (LTPs) are lipid-binding allergens whose natural ligands are not fully known. To elucidate the function and allergenic relevance of nsLTP-lipid complexes, purified natural *Lupinus luteus* (*L. luteus*) nLTP and recombinant peach LTP, rPru p 3, were tested for membrane interaction and lipid transport activity using liposome assays and Förster-resonance-energy-transfer (FRET) in a case-level proof-of-principle investigation. Allergenic relevance of the LTP-lipid interaction was evaluated in the presence of oleic acid (OA), phosphatidylcholine (PC), phosphatidylglycerol (PG), and phosphatidylserine (PS) in a basophil activation test (BAT) with effector cells from an LTP-allergic patient. Both LTPs interacted with neutral PC and negatively charged PS liposomal membranes. A novel transport activity for anionic PG species was identified for both proteins, indicating a shared functional preference for the glycerol headgroup. LTP-dependent lipid exchange/mixing were consistent with transfer. However, fusion/mixing mechanisms cannot be excluded with the current readout. In BAT, both LTPs showed enhanced activation in combination with OA, PC, PG, and PS. As PG is a key component of bacterial membranes, the PG specificity of the lipid interaction of *L. luteus* nLTP and rPru p 3 is likely of relevance in allergen interaction with the gut microbiome and for enhancement of allergic symptoms. These findings highlight lipid-specific functional properties and lipid-dependent modulation of allergenic activity in plant nsLTPs.

**Supplementary Information:**

The online version contains supplementary material available at 10.1038/s41598-026-55182-9.

## Introduction

Non-specific (ns) lipid transfer proteins (LTPs) are lipid-binding allergens and relevant causes of allergic symptoms in sensitized individuals. The clinical symptoms to nsLTPs range from mild and moderate - like the oral allergy syndrome - to severe reactions and anaphylaxis, the reason for the latter being hypothesized to be due to their lipid-binding capacity. nsLTPs are expressed in various plant food sources. Especially fruits of the *Rosaceae* family such as cherry, peach, and apple contain high amounts of LTP, however, many more nsLTP-containing plant-foods also belong to the nut group and to cereals (wheat, maize and rice)^1^. Until only a few years ago, nsLTP-associated allergic reactions were mainly reported in patients in the Mediterranean area^1^, the primary sensitizer being peach and the exposure to the relevant LTP Pru p 3 from *Prunus persica* via consumption^1,2^. However, there is increasing evidence that nsLTPs are responsible for anaphylaxis in Northern Europe as well^3–5^. LTPs are highly cross-reactive allergens and induce the so-called LTP syndrome in patients, i.e. allergic responses to many nsLTPs from different sources often belonging to very distant species^6^. The allergenicity of nsLTPs is driven by their molecular characteristics. They are small, abundant, and soluble, heat- and digestion-resistant proteins that are able to bind and transfer different lipids between membranes in vitro^7^. Class I nsLTPs are characterized by a hydrophobic cavity that is formed by four intramolecular disulfide bridges based on a common motif of eight cysteines within the protein sequence. This hydrophobic cavity pervades the molecule with a tunnel-like structure^1^. The term “non-specific” indicates that a variety of lipids is able to interact with nsLTPs, for example fatty acyls, glycerolipids, sterol lipids, glycerophospholipids, sphingolipids, prenol lipids, saccharolipids, and polyketides^8,9^. nsLTPs differ regarding their lipid binding capacities: some can bind up to two lipids simultaneously, some are incapable of binding and transporting free lipids or do not have the structural predisposition like the hydrophobic tunnel-like structure where lipids are bound and transported^10^. The detailed role of the allergen-lipid interaction in the development of allergic diseases contains considerable gaps^11^. Available studies have addressed nsLTPs from different plant sources, and the data on nsLTP lipid ligands are somewhat contradictory regarding allergenicity and allergy protection. Dubiela and co-workers demonstrated that the epitopes from Pru p 3 were modified in the presence of free fatty acids (“structural plasticity”), leading to higher IgE binding in the presence of oleic acid (OA)^12^. Later, they demonstrated a similar effect for the walnut LTP Jug r 3^13^. However, in the case of lentil nsLTP (Len c 3), oleic acid did neither increase nor reduce IgE binding^14^. The LTP of tomato seeds, Sola l 7, is associated with anaphylactic reactions after tomato ingestion, although it is considered a minor allergen in the whole tomato fruit^15^. A protein-biochemical analysis employing NMR analysis of lipid-interaction with tomato nsLTP rSola l 7 revealed that unsaturated oleic and linoleic fatty acids presented higher affinity to the allergen, subsequently inducing more significant changes than saturated or short-chain fatty acids which suggested epitope modification after this interaction^16^. Investigations of the effect on allergenicity using patients` IgG and IgE antibodies in the presence of free fatty acids revealed a significantly lower antibody binding than in the absence of the lipids, an observation, that the authors interpreted as suggestive for a potential protective role of unsaturated fatty acids in tomato allergy^16^.

There is increasing evidence that nsLTPs cause severe allergic reactions in patients in Northern Europe^3,4^ in spite of the fact that in this geographical area the main sensitizer is the major birch pollen allergen, Bet v 1^17^. Since there is an increasing consumption of vegetarian/vegan food (clinical relevance) and still a gap in knowledge as to the detailed mechanistic effects of LTPs in combination with their lipid ligands our proof-of-principle study aimed at investigating the lipid-binding and membrane-interacting potential of a newly purified LTP from the legume yellow lupine (*Lupinus luteus*) and the effect of allergen-lipid-interaction on its allergenicity.

## Results

### Membrane interaction of naturally purified *L. luteus* LTP (*L. luteus* nLTP)

**Fig. 1:**
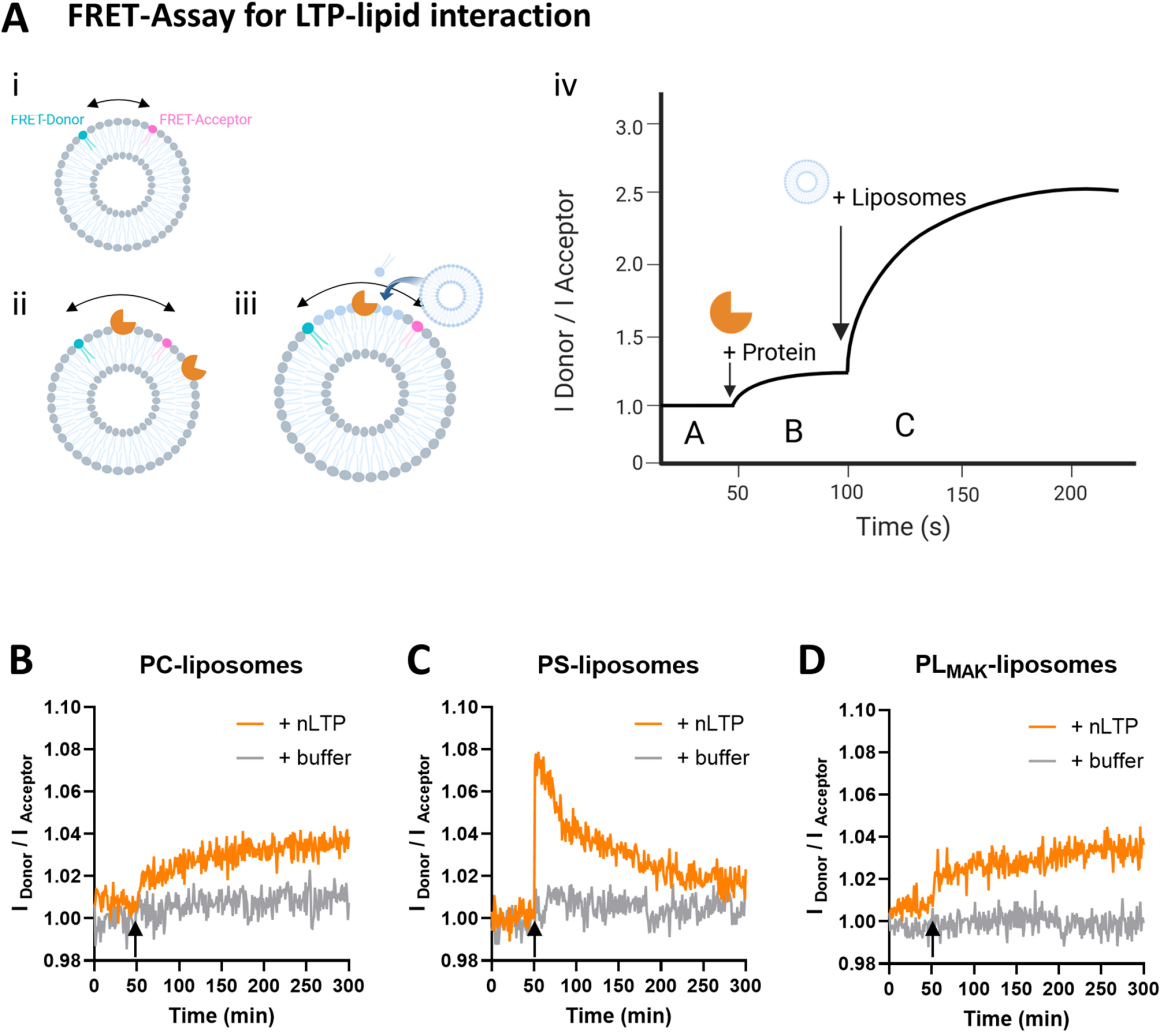
(**A**–**D**) Membrane interaction of purified *L. luteus* nLTP. (**A**) (i) Principle of Förster-resonance-energy-transfer (FRET)-assay on liposomes (ii) to study the membrane intercalation of protein (orange symbol) after addition of LTP to labelled liposomes and (iii) to study lipid-transport activity of LTP after addition of unlabelled liposomes into the assay. (iv) A schematic measurement profile is indicated. Measurements were performed in physiological buffer at a constant temperature of 37 °C. (Graph A, i-iv created with BioRender software by A.B.S). Changes in the membrane surface of liposomes were analyzed by FRET-assay, liposomes made from PC, PS, or a lipid mixture resembling the phospholipid composition for macrophages PL_MAK_. The liposomal bilayer was labeled with the FRET dyes *NBD-PE (donor) and *Rh-DHPE (acceptor) at a ratio 100:1:1 M. The labeled liposomes (**B**) PC**, (**C**) PS**, and (**D**) PL_MAK_** were diluted to 10 µM in 20 mM HEPES, 150 mM NaCl, pH 7.4 and the signals of donor and acceptor adjusted to equal values of intensity. After baseline recording for 50 s, *L. luteus* nLTP protein or buffer volume control was added to a final concentration of 10 µg/mL at time point 50 s and the signals recorded until 300 s. Ratios I_Donor_/I_Acceptor_ were calculated, and representative data of *n* = 2 independent measurements is shown.

To study the lipid interaction of *L. luteus* nLTP, we employed phospholipid liposomes of different compositions allowing to investigate the specificity of membrane interaction. Labelling of the liposomes with Förster-resonance-energy-transfer (FRET)-dyes provides a highly sensitive method to study changes in the membrane surface area by the integration of proteins or lipids into the membrane bilayer, a fact that is of particular importance for the investigation of small compounds like the purified *L. luteus* nLTP. The FRET-assay was performed as a probe-dilution-assay as depicted in the experimental scheme (Fig. 1A) to study membrane interaction of the allergen. In a two-step assay, the lipid transport function of the allergen was investigated by offering unlabelled liposomes and measuring the integration of lipids into the FRET-sensitive target membrane. Different lipid compositions of the liposomes were chosen to reveal interactions with phosphatidylcholine (PC), the major phospholipid in all eukaryotic cells, phosphatidylserine (PS), as a prototypic member of a negatively charged phospholipid, and a lipid mixture resembling the lipid composition of a macrophage membrane (PLMAK) as described by us earlier^18^, to analyse potential interaction or interference of *L. luteus* nLTP with immune cells participating in the pro-inflammatory phase of allergic reactions. Addition of *L. luteus* nLTP to the liposomes induced an increase of the FRET signal ratio as observed when proteins or peptides integrate into the phospholipid bilayer or when protein membrane association causes an increase in lipid headgroup spacing. The membrane interaction of *L. luteus* nLTP occurred fast and resulted in a stable signal ratio over time in PC and PLMAK liposomes (Fig. 1B, D). In PS liposomes, a biphasic reaction was observed with an initial strong signal change, followed by a soft decline into a steady state after 50–100 s (Fig. 1C), a behaviour that could indicate reorganization of the membrane upon the protein interaction.

**Fig. 2:**
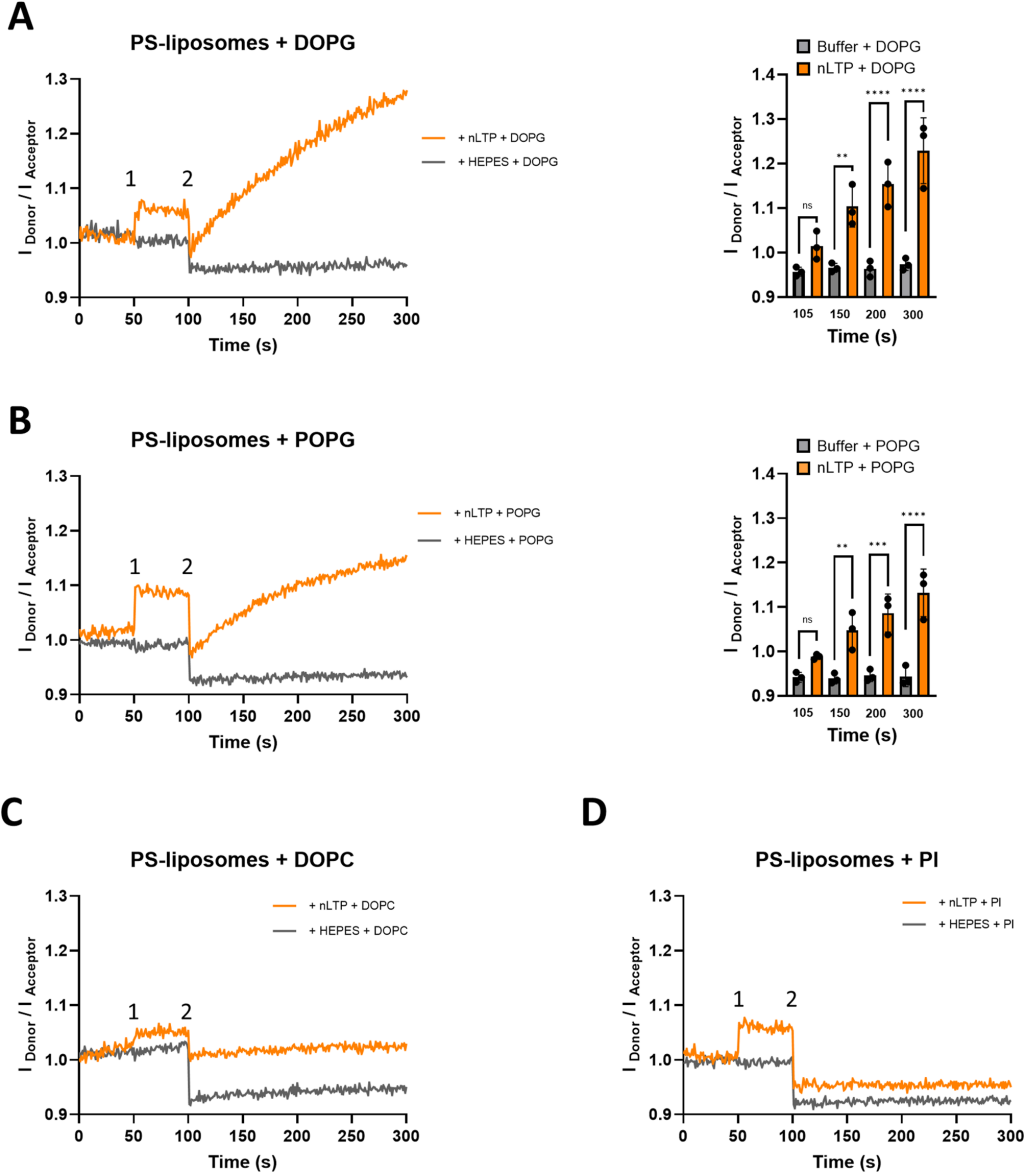
(**A**–**D**) Lipid transport activity of purified *L. luteus* nLTP, abbreviated as nLTP. Changes in the membrane surface of liposomes were analyzed by a Förster-resonance-energy-transfer (FRET)-assay according to the model Fig. 1A. Liposomes made from PS were labeled with the FRET dyes *NBD-PE (donor) and *Rh-DHPE (acceptor) at a ratio 100:1:1 M. The labeled liposomes were diluted to 10 µM in 20 mM HEPES, 150 mM NaCl, pH 7.4 and the signals of donor and acceptor adjusted to equal values of intensity. After baseline recording for 50 s, nLTP protein (orange line) or buffer control (grey line) was added to a final concentration of 10 µg/mL at time point 50 s (addition 1). Unlabeled liposomes composed of (**A**) DOPG, (**B**) POPG, (**C**) DOPC, or (**D**) PI were added at time point 100 s (addition 2) to a final molar ratio of 10 µM and the signals recorded until 300 s. Curves show a representative data set, bar graphs show mean ± SD of *n* = 3 independent measurements. Data were analyzed by ordinary one-way ANOVA with Bonferroni’s post-test for multiple comparisons of samples, significance compared to buffer control is indicated * ≤ 0.05; ** ≤ 0.01; *** ≤ 0.001; ****≤ 0.0001; ns, not significant. For explanation: addition 1 corresponds with part B in Fig. 1A iv, indicating the intercalation of the LTP into the membrane surface, while addition 2, corresponding with part C in Fig. 1A iv, represents the directed transport of phospholipid molecules into the labeled liposomes.

For functional analysis of *L. luteus* nLTP, a lipid transport assay was performed on PS** liposomes. PS** liposomes were first incubated with *L. luteus* nLTP and in a second step exposed to unlabelled phospholipid liposomes at equimolar ratio. Addition of dioleoyl-sn-glycero-3-phospho-(1’-rac-glycerol) (DOPG) or palmitoyl-2-oleoyl-sn-glycero-3-phospho-(1’-rac-glycerol) (POPG) both induced a time dependent change of the FRET signal ratio with a notable but not statistically significant increase after 5 s and highly significant increases compared to control in the absence of *L. luteus* nLTP after 50, 100 and 200 s for DOPG and POPG by transport of lipids into the target PS** liposomes (Fig. 2A, B). Kinetics and change in the FRET ratio were comparable for both PG species. In contrast, exposure to neutral PC liposomes did not show any transport activity, neither did the exposure to negatively charged phosphatidylinositol (PI) liposomes (Fig. 2C, D), indicating a preference for the interaction of *L. luteus* nLTP with the phosphatidylglycerol headgroup. Of note, addition of unlabelled DOPG or POPG liposomes to PS** liposomes did not lead to any increase of the FRET signal ratio in the absence of *L. luteus* nLTP, demonstrating that in the absence of the nLTP no lipid mixing or fusion of liposomes occurred. LTP-dependent lipid exchange/mixing were consistent with transfer. However, fusion/mixing mechanisms cannot be excluded with the current readout.

**Fig. 3:**
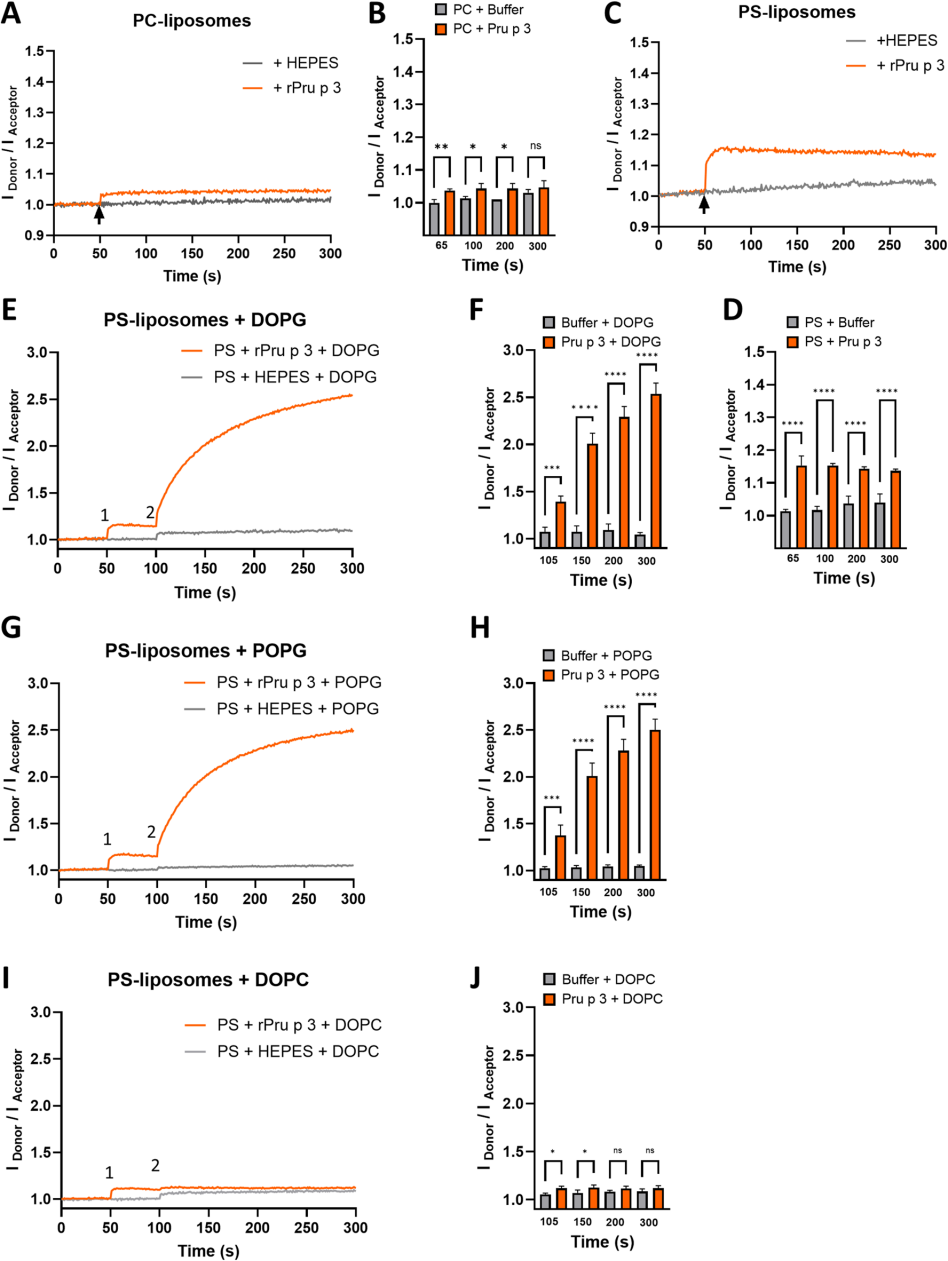
(**A–J**) Membrane activity and lipid transport activity of recombinant Pru p 3. Changes in the membrane surface of liposomes were analyzed by a Förster-resonance-energy-transfer (FRET)-assay according to the model Fig. 1A. Liposomes made from (**A**, **B**) PC or (**C**, **D**) PS were labeled with the FRET dyes *NBD-PE (donor) and *Rh-DHPE (acceptor) at a ratio 100:1:1 M. The labeled liposomes PC** or PS** were diluted to 10 µM in 20 mM HEPES, 150 mM NaCl, pH 7.4 and the signals of donor and acceptor adjusted to equal values of intensity. After baseline recording for 50 s, rPru p 3 (orange line) or buffer control (grey line) was added to a final concentration of 10 µg/mL at time point 50 s. (**E**–**J**) For analysis of lipid transport activity, (1) rPru p 3 (orange line) or buffer control (grey line) was added to PS** liposomes at time point 50 s (addition 1), and (2) unlabeled liposomes composed of (E) DOPG, (G) POPG, or (I) DOPC were added at time point 100 s (addition 2) to a final molar ratio of 10 µM and the signals recorded until 300 s. Line graphs show mean, bar graphs mean and SD of *n* = 3 independent measurements. (B, D, F, H, J) Data were analyzed at the indicated time points by ordinary one-way ANOVA with Bonferroni’s post-test for multiple comparisons, significance compared to buffer control is indicated * ≤ 0.05; ** ≤ 0.01; *** ≤ 0.001; **** ≤ 0.0001; ns, not significant. For explanation: addition 1 corresponds with part B in Fig. 1A iv, indicating the intercalation of the LTP into the membrane surface, while addition 2, corresponding with part C in Fig. 1A iv, represents the directed transport of phospholipid molecules into the labeled liposomes.

The purification of hydrophobic and lipid binding allergens from natural sources still poses an experimental challenge and requires refinement to provide sufficient amounts of material for structural and functional investigation. We, therefore, chose to include the LTP Pru p 3 from peach for further analysis because n/rPru p 3 and lipid interactions have been investigated with lauric acid, cis-parinaric acid, palmitic acid, and linoleic acid^12^ and their effect on IgE-binding was subsequently shown. In our study, rPru p 3 showed fast and stable integration into PC liposomes, with a reproducible moderate but significant increase in FRET ratio at time points 15s, 50s, and 100s after addition (Fig. 3A, B). In PS liposomes, a strong integration of rPru p 3 was followed by a slow decline of the FRET ratio resulting in a significant change (*p* ≤ 0.0001) of the FRET ratio at 300 s (Fig. 3C, D). Analysis of transport activity of rPru p 3 in PS** liposomes showed a strong time-dependent increase of the FRET ratio by exposure to unlabelled liposomes composed of DOPG and POPG, with highly significant changes (*p* ≤ 0.0001 for DOPG, *p* ≤ 0.0001 for POPG at time point 300s) indicating efficient transport activity (Fig. 3E–H), whereas exposure to PC liposomes did not indicate a major transport activity for the zwitterionic neutral phospholipid beyond a small, but statistically significant increase observed at 5s and 50s after liposome addition (Fig. 3I, J).

### Basophil activation by *L. luteus* nLTP and rPru p 3 in the presence of lipids

**Fig. 4:**
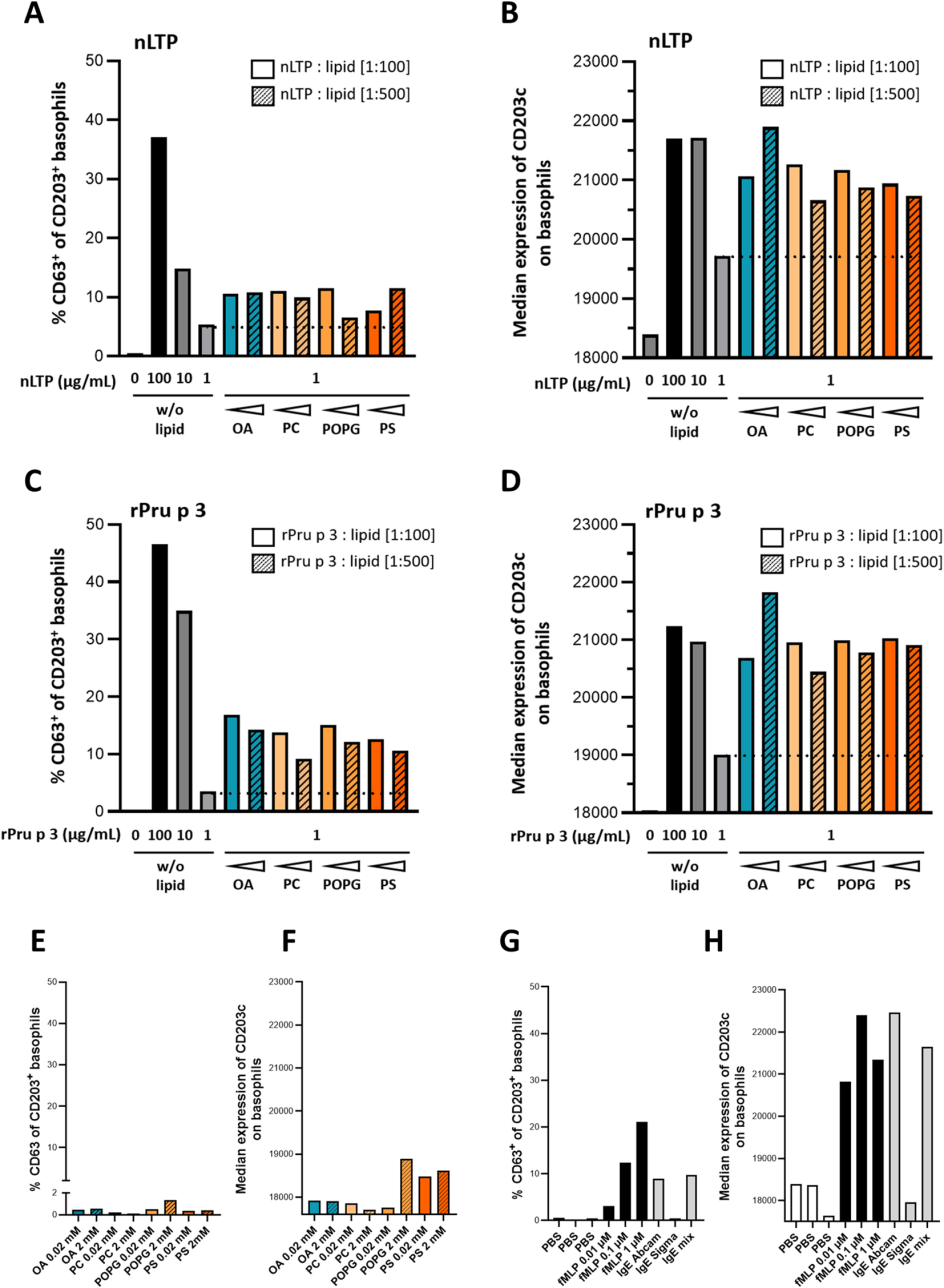
(**A–H**) Lipids enhance basophils activation by *L. luteus* nLTP and rPru p 3. Basophil activation test (BAT, flow cytometric readout, gating strategy^19^ shown in Fig. S1) with whole blood from an LTP-allergic patient using *L. luteus* nLTP (abbreviated as nLTP) (**A**, **B**) and rPru p 3 (**C**, **D**), tested alone or in combination with lipids (oleic acid [OA], phosphatidylcholine [PC], 1-palmitoyl-2-oleoylphosphatidylglycerol [POPG], and phosphatidylserine [PS]). Filled bar charts: 1:100; hatched bar charts: 1:500 molar ratio. Controls with lipids only (**E**, **F**) showed no activation (**E**; minimal CD63 expression on CD203c^+^ basophils) or only minor CD203c upregulation with PS (**F**), comparable to negative controls (H, PBS). Positive controls (**G**, **H**; fMLP and anti-IgE) confirmed BAT functionality, with increased CD63^+^ basophils (**G**) or elevated CD203c expression (**H**, indicated by higher median fluorescence intensity). Dotted lines in panels A–D indicate the level of stimulation with nLTP or rPru p 3 (1 µg/ml) without added lipids. Analysis of basophils of one representative *L. luteus* nLTP allergic patient. Statistical analysis not applicable.

Food-derived nsLTP allergens may encounter lipids in the originating plant tissue, during food preparation and processing, and in the gastrointestinal tract after ingestion. Thus, the likelihood of allergen-phospholipid interaction is high. We assessed the impact of lipids on immunological responses in a basophil activation test (BAT). Basophil activation, mediated by allergen-specific IgE in the patient’s serum, is indicated by expression of the surface marker CD63. Analysis of the allergen response in blood derived from a 37-year-old lupine-allergic female patient showed an increase of CD63^+^ activated basophils from < 0.5% in the unstimulated control up to 5.4% after exposure to 1 µg/mL *L. luteus* nLTP and a dose-dependent increase (Fig. 4A, 10 µg/mL *L. luteus* nLTP = 14.9%, 100 µg/mL *L. luteus* nLTP = 37.1%). Stimulation with 1 µg/mL *L. luteus* nLTP activated 5.4% of basophils which nearly doubled to ~ 10% (9.98% mean increase) in the presence of OA, PC, POPG, or PS. This level of activation corresponded to ~ 75% of that induced by a tenfold higher concentration (10 µg/mL) of *L. luteus* nLTP. Consistently, CD203c expression on basophils induced by 1 µg/mL *L. luteus* nLTP was also enhanced by the presence of these lipids (Fig. 4B). Similar results were obtained with rPru p 3, an nsLTP commonly used in allergy diagnostics and known for cross-reactivity. Basophil activation increased from 3.5% with 1 µg/mL rPru p 3 alone to ~ 13–15% in the presence of OA, PC, DOPG, or PS (Fig. 4C), accompanied by maximal CD203c upregulation (Fig. 4D). The magnitude of activation (CD63 and CD203c) was comparable to that induced by positive control stimuli (fMLP and anti-IgE; Fig. 4G, H). In contrast, lipid compounds alone were inert in BAT, with only minor background effects on CD203c expression (Fig. 4E, F).

## Discussion

This is the first case-level proof-of-principle investigation of the membrane interaction of a novel lipophilic allergen, the LTP from yellow lupine (*L. luteus*) in its natural form, which has recently been shown to activate basophils from a patient with LTP syndrome^4^, a severe allergic disease. Yellow lupine seeds are increasingly used for food as new dietary alternative and are strongly acknowledged by individuals applying vegetarian or vegan diet or people who are allergic to milk or wheat products^20^. Their components are of high nutritional value including a favourable protein/fat ratio. As additional advantage for allergic individuals, they are gluten- and lactose-free^21^. Since many allergens of lipophilic nature are associated with more severe allergic reactions, lipid-binding capacity and the effects of allergen-lipid-interaction—apart from protein protection from heat and digestion^12,16,22^—are of elementary interest in research on sensitizing and allergy eliciting processes^23^.

Structural studies on lipid-LTP interaction have revealed that some lipids increase the IgE-binding capacity by altering the “tunnel”-like LTP cavity (“structural plasticity”)^12^. Molecular investigation of Pru p 3 showed that binding of OA to the hydrophobic cavity induced conformational changes exposing the major IgE epitope of Pru p 3, which is responsible for severe reactions. OA binding was also confirmed for the recombinant (r) variant of Pru p 3, which is highly relevant to the present investigation, as rPru p 3 served as a key comparator molecule. Similar results were obtained for the walnut-LTP Jug r 3: OA affected the IgE-binding capacity of rJug r 3 in an IgE-ELISA by increasing it up to 7.5 fold, due to additional IgE-epitopes exposed via structural plasticity, and the structural prerequisites for lipid-binding preferences (longer-chain and unsaturated fatty acids) were shown to be similar to Pru p 3^13^. Structural investigations extended to further food LTPs with differences regarding their allergenic potential: Mal d 3 (apple), Cor a 8 (hazelnut), and Hel a 3 (sunflower seed), the potential decreasing in that order^24^. All LTPs had the highest preference to OA when compared to other lipid ligands^24^, which was confirmed for Pru p 3 in the study presented here and was observed for yellow lupine LTP as well. Aina et al. showed that among OA, stearic and lauric acids, OA was the most effective concerning the induction of changes of the molecular structure by increasing the cavity volume, thereby affecting the protein conformation with a subsequent increase in IgE-binding. That was observed for Mal d 3 and to a smaller extent for Cor a 8^24^. In our study, no antibody-based assays were applied. Instead, BAT was used to investigate the effect of lipid ligands on the intensity in which the LTPs were able to activate basophils, a reaction that can be IgE-mediated but is not necessarily exclusively IgE-mediated. In BAT, OA, PC, POPG, and PS in combination with lupine and peach LTP induced an enhanced basophil activation. The results suggest an increase in IgE-binding capability due to structural changes, since Dubiela and co-workers as well as Aina and co-workers provided strong data to this effect for relevant food LTPs and OA previously. In contrast to these research teams, we included other lipid ligands in addition to OA: PC, PG, POPG, and PS^12,24^.

A detailed lipidomics analysis of *L. luteus* seeds identified phosphatidylcholine (PC) as dominating lipid species with about 41%, followed by 30% lyso-PC species, 13% phosphatidylethanolamines, and about 5% PGs^25^.

The here reported transport activity of *L. luteus* nLTP and rPru p 3 for anionic PG indicates a common lipid specificity. PG lipid species express a negative surface charge available for electrostatic interactions. In a previous study, we have reported the zeta-potential of DOPG and POPG liposomes in phosphate buffered saline in the range of − 80 mV^26^. Interestingly, the data presented here strongly indicate that the transport activity of *L. luteus* nLTP and rPru p 3 has a preference for the glycerol headgroup rather than being driven solely by electrostatic interactions, as the negatively charged PI did not interact with the LTPs. In addition to the previously mentioned structure-based analyses of allergen-lipid interactions, this unique application of FRET allowed an investigation of soluble LTP - lipid membrane interaction under near-physiological conditions. In addition, the FRET assay provides high sensitivity for detection of transport activity and lipid selectivity of LTPs, thereby showing LTP functionality other than allergenicity.

In the context of food allergens, several sources of lipids for lipid-allergen interaction are conceivable, which are outlined in the following. In plants, PG lipids are a major and essential component in the thylakoid membrane of chloroplasts^27–29^, and involvement in plant and seed lipid metabolism has been suggested^30^. However, as for many of the plant food LTPs, their physiologic role in plant cells or seeds has not been clarified in detail. In most mammalian tissues and biological fluids, PG lipids are relatively scarce. However, the human gut microbiome constitutes a rich source of microbial lipids, including PG lipid species. In Gram-negative bacteria, PG constitutes for 20–25% of the lipids in the cell-envelope, in Gram-positive bacteria PG makes up to 60% of cell wall lipids. Ligand interaction of food LTPs with PG lipids in the gastrointestinal tract thus presents a reasonable scenario that should be considered as a sensitization route. Recent lipidomics studies demonstrate the PG lipid diversity in human gut bacteria and suggest that microbiome composition and diet may shape both, the amount and the spectrum of PG lipid species in the human gut^31,32^.

Another organ with abundant PG lipid species is the lung. PG is a significant component of pulmonary surfactant, accounts for approximately 7–15% of total phospholipids, and plays a major role as a crucial immune regulator in the lung^33,34^. A recent study indicates a major impact of the pulmonary route for sensitization and symptoms related to LTP^35^. Interaction of inhaled food allergens with PG in pulmonary surfactant needs to be considered particularly for the here investigated allergen *L. luteus* nLTP since occupational asthma following work with lupine flour occurs^21,36^.

Following allergen-(lipid)-barrier interaction, allergen induced inflammation is the second arm of allergic response contributing to symptoms and pathology in acute and chronic allergies. Macrophages are major drivers of inflammation with a particular role in pathogen detection, inflammation, and immune defense. Their recognition of bacterial ligands such as lipopolysaccharide (LPS, endotoxin) can trigger TLR4/MD-2 and TLR4-independent intracellular LPS receptors such as cytoplasmatic caspases-1, -4, and -5. This recognition is enhanced by LPS-binding protein (LBP)^37^. This LTP can transport LPS to enhance its recognition by cellular receptors. A promiscuous interaction with anionic phospholipids has been described^38,39^, and we have recently demonstrated that anionic lipids such as phosphatidylglycerol species can interfere with LPS induced activation of both pathways in vitro and in vivo^26,40,41^. Of note, our data on the interaction of *L. luteus* nLTP with the macrophage model membrane is a strong indicator for immune cell interaction reported here for the first time and will be addressed in future studies.

Due to contradictory reports on the effects of allergen-lipid-binding on allergenicity of LTPs from different foods, we analyzed the new *L. luteus* nLTP and rPru p 3 in combination with four lipid components in BAT with cells from a patient with LTP syndrome. The data revealed an amplifying effect of the lipids on basophil activation for both LTPs. This effect was pronounced for the recombinant Pru p 3 and moderate for the new lupine nLTP, likely because the lipophilic cavity of the natural LTP variant is already occupied by endogenous lipids. Although we did not observe a major lipid transport activity for PC, both allergens, lupine nLTP and rPru p 3 induced a moderate but clearly observable and statistically significant interaction with the surface of PC liposomes. This membrane association or an intercalation of a hydrophobic region of the protein might indeed be sufficient to induce changes in the protein conformation or its presentation and might explain the enhancing biological effect as observed also for PC liposomes in the BAT. Thus, the transport activity indicates a preference of the nLTP and rPru p 3 for certain lipids, such as revealed for phosphatidylglycerol. But also weaker lipid interactions as such observed for the neutral PC can affect the allergic response.

Our single patient analysis provides first proof-of-principle data on a combination of new biophysical test results and an optimized cell-based allergy detection method, confirming the allergenic effect of the LTP alone and demonstrating furthermore its amplification by certain lipids revealing a probable sensitization mechanism. The observed allergic amplification effect underscores the need for targeted patient identification for further testing. The purification of sufficient amounts of natural purified LTP, however, still poses a challenge and limits their experimental analysis. Development of advanced purification protocols will be a future task for these studies.

In conclusion, both purified lupine and recombinant peach LTP are allergenic and active in BAT. Natural *L. luteus* LTP rapidly interacts with membranes, and lipid transport assays demonstrated preferential effects with PG under these conditions. Effects of certain lipid compounds (OA, neutral lipid PC, negatively charged lipids PS, and PG), were found to amplify LTP allergenicity in the BAT with one, though representative, patient in this case-level proof-of-principle investigation and will have to be confirmed with a larger number of LTP- and lupine-allergic patients. The observed functional activity and preference for PG lipids, which are abundantly present in the gut and the lungs – may be indicative for an increased risk of lipid-mediated exacerbation of allergic symptoms.

## Materials and methods

The study protocol was reviewed and approved by the ethics committee of the University of Lübeck, Germany, approval ID 10–124. The patient whose basophils were analyzed provided informed consent in writing prior to her enrollment in the study, and the study was conducted in accordance with the Declaration of Helsinki. The 37-year-old female patient suffered from multiple food allergies (peach, hazelnut, walnut, apple, but not peanut) due to cross-reactivity via LTPs, known as LTP syndrome, and had specific IgE to the plant food sources lupine seed (*Lupinus albus*), apple, walnut, and peanut^4^. In vitro allergy diagnostics revealed positive reactions to six food nsLTPs in descending order: peach (rPru p 3), apple (rMal d 3), walnut (rJug r 3), peanut (rAra h 9), wheat (rTri a 14), hazelnut (rCor a 8) with IgE in ImmunoCAP (Thermo Fisher Scientific, Freiburg, Germany and Uppsala, Sweden). She also reacted to *L. luteus* nLTP (nLTP), recently described by our group^42^, as shown by immunoblot analysis. This new allergen was also able to activate her basophils^4^, providing the rationale for the present study using this new LTP (*L. luteus* nLTP) and the patient’s basophils. All methods were performed in accordance with the relevant guidelines and regulations.

### *Lupinus luteus* LTP extraction from the natural source (*L. luteus* nLTP)

Lupine extracts were produced from dry seeds of *Lupinus luteus*, var. Juno using two extraction protocols for acidic and alkaline protein extraction, respectively. Subsequent allergen identification and characterization was performed as previously described^42^. Since low molecular weight (LMW) allergens have been detected by patients’ sera in the acidic extracts, allergen purification was performed with the acidic extracts, focussing on *L. luteus*. This procedure yielded a low molecular weight protein of about 10 kDa. This protein was analysed by immunoblot, isolated, further purified, and subsequently confirmed as an LTP by N-terminal sequencing and mass spectrometric analysis^42^.

### Membrane interaction of naturally purified *L. luteus* LTP (*L. luteus* nLTP)

Förster-resonance-energy-transfer (FRET) on liposomal membranes was employed to investigate the nLTP newly purified from *L. luteus* as well as the recombinant allergen Pru p 3 (Indoor Bio, USA) as a control LTP with already known lipid-binding capacity^8^ for lipid interactions. This method detects molecular interactions with high spatial sensitivity in the Å-range. Phospholipid liposomes were labeled in the membrane headgroup area with a pair of FRET-dyes, enabling the analysis of membrane interaction and transport function of the LTP protein in solution in a physiological buffer system without the need of labeling the protein.

### Lipids and reagents for model membrane analysis

Lipids used in this study are specified in Table 1. Chicken egg L-α-phosphatidylcholine (PC), bovine liver L-α-phosphatidylethanolamine (PE), bovine liver L-α-phosphatidylinositol (PI), porcine brain L-α-phosphatidylserine (PS), porcine brain sphingomyelin (SM), DOPC [18:1-(Δ9-*cis*)-PC, 1,2-Di-(9Z-octadecenoyl)-*sn*-glycero-3-phosphocholin sodium salt], DOPG [18:1-(Δ9-*cis*)-PG, 1,2-dioleoyl-*sn*-glycero-3-phospho-(1’-*rac*-glycerol) sodium salt], and POPG [16:0/18:1-PG, 1-palmitoyl-2-oleoyl-*sn*-glycero-3-phospho-(1’-*rac*-glycerol) sodium salt] were purchased from Avanti Polar Lipids. Oleic acid [C18:1] (OA) was from AppliChem (Darmstadt, Germany). N-(7-nitrobenz-2-Oxa-1,3-diazol-4-yl)-1,2-dihexadecanoyl-sn-glycero-3-phosphoethanolamine (NBD-PE) and Lissamine rhodamine B 1,2-dihexadecanoyl-sn-glycero-3-phosphoethanolamine (Rh-DHPE) were from Invitrogen, Molecular Probes (Eugene, OR, USA).

**Table 1:** Lipids and respective abbreviations used in this investigation of LTP-lipid-interaction, transport function, and basophil activation test (BAT).

Abbreviation	Lipid	Source
PC	L-α-phosphatidylcholine	Chicken egg
PE	L-α-phosphatidylethanolamine	Bovine liver
PI	L-α-phosphatidylinositol	Bovine liver
PS	L-α-phosphatidylserine	Porcine brain
SM	sphingomyelin	Porcine brain
DOPC	[18:1-(Δ9-*cis*)-PC, 1,2-Di-(9Z-octadecenoyl)-*sn*-glycero-3-phosphocholin sodium salt]	Synthetic
DOPG	[18:1-(Δ9-*cis*)-PG, 1,2-dioleoyl-*sn*-glycero-3-phospho-(1’-*rac*-glycerol) sodium salt]	Synthetic
POPG	[16:0/18:1-PG, 1-palmitoyl-2-oleoyl-*sn*-glycero-3-phospho-(1’-*rac*-glycerol) sodium salt]	Synthetic
OA	Oleic acid [C18:1]	Eukaryotic

### Liposome preparation

Liposomes were prepared from chloroform stocks, either from the single phospholipids PC, PS, PI, DOPC, DOPG, and POPG, or from a lipid mixture resembling the composition of macrophage membranes (PL_MAK_) [PC: PS: PE: SM] = 1:0.4:0.7:0.5 M. Target liposomes for FRET-analysis were labeled with *NBD-PE and *Rh-DHPE at a molar ratio of [lipids: NBP-PE: Rh-DHP] = 100:1:1 M. The organic solvent was evaporated under a stream of nitrogen until completely dry. Lipids were then hydrated in 20 mM HEPES, 150 mM NaCl, pH 7.4 (for FRET assay) or in PBS (for BAT) to a final concentration of 1 mM or 10 mM, respectively. Liposome formation was induced by pulsed ultrasound (Ultrasonic-Homogenizer HTU Soni130, 1 min, pulse on/off: 2 s, amplitude 30%) followed by three rounds of thermocycling between 4 °C and 56 °C for 30 min each. Preparations were stored overnight at 4 °C before use.

### Förster-resonance-energy-transfer (FRET) assay

FRET experiments were performed on phospholipid liposomes double-labeled with *NBD-PE (donor) and *Rh-DHPE (acceptor) (PC**, PS**, or PL_MAK_**). The assay was performed as a probe-dilution assay, i.e. integration of protein or phospholipids into the labeled liposomes lead to an increase of the membrane surface area, indicated by an increase of the signal ratio I_Donor_/I_Acceptor_^39^. For measurements, labeled liposomes were diluted to 10 µM, the signal ratio I_Donor_/I _Acceptor_ was adjusted to 1, and the baseline was recorded. At the indicated time point (50 s) protein or buffer control was added. For the analysis of lipid transport activity, at time point 50 s protein was added to the liposomes, at time point 100 s unlabeled liposomes were added to the target liposomes. Recordings were performed at 37 °C under constant stirring on a Fluorolog-3 (Jobin Yvon Inc., Edison, NJ, USA).

### Basophil activation test (BAT)

The following ligands were chosen for the investigation of the potential increase of LTP allergenicity in BAT: oleic acid (OA), phosphatidylcholine (PC), 1-palitoyl-2-oleoylphosphatidylglycerol (POPG), phosphatidylserine (PS). Lipids were chosen as prototypic major phospholipids of eukaryotic membranes (neutral PC, negatively charged PS), or bacterial membranes (PG), which LTPs may be exposed to in the gastrointestinal tract. OA is a known ligand of Pru p 3, Jug r 3, maize LTP and others^13^. PC and Lyso-PC, as well as PG and lyso-PG lipid species have been shown to be ligands for a number of food LTPs including wheat LTP1, wheat LTP2, pea LTP, and tomato LTP^8^. As an LTP comparator, we chose rPru p 3 because it has been investigated in detail for its lipid-association and shown to have a strong lipid-binding capacity^8^ affecting its allergenicity. In addition, it is considered the clinically most important and best characterized food LTP and a marker allergen for LTP-sensitization in general. The new *L. luteus* nLTP has been proven previously to be allergenic^4^, but requires further functional and immunological analysis.

*L. luteus* nLTP and rPru p 3 were used as allergens for preincubation (15 min at 37 °C) with lipid liposomes (diluted from 10 mM stocks in PBS) in solution (Table 1) and subsequent basophil stimulation (30 min at 37 °C) with lipid-preincubated allergen and respective controls. Specifically, rPru p 3 was used from three different stock concentrations (100, 10, and 1 µg/ml). Lipids (OA, PC, POPG, PS) were diluted in PBS (Pan Biotech, Aidenbach, Germany) to a molar ratio of 1:100 and 1:500 (allergen to lipid). Similar concentrations and dilutions were used for nLTP, taking account for the allergen-lipid ratio. *L. luteus* nLTP concentration 0,15 µg/ml, diluted 1:1.5, 86.7 µl *L. luteus* nLTP + 43.4 µl PBS (up to 100 µg/ml). rPru p 3 (concentration 1.2 mg/ml, diluted 1:12), 10.9 µl rPru p 3 + 119.1 µl PBS (100 µg/mL). Samples were prepared accordingly: 10 µL PBS, 10 µl LTP and 10 µl of the respective lipid per well of a 96-well plate (Sarstedt AG & Co. KG, Nümbrecht, Germany). The samples were incubated for 15 min at 37 °C before stimulation of basophils. The stimuli formyl-methionyl-leucyl phenylalanine (fMLP 1µM, Sigma-Aldrich, Steinheim, Germany), anti-IgE (1:1 mixture of goat anti-human IgE, 1 µg/ml, Sigma-Aldrich, Steinheim, Germany, and goat anti-human IgE, 1 µg/ml, Abcam, Cambridge, UK) were used as positive controls for the BAT, PBS (Sigma-Aldrich, Steinheim, Germany) for dilutions and as negative control.

#### Flow cytometric instruments and antibodies

Measurements were conducted on a FACSymphony A1 flow cytometric instrument (BD Biosciences, San Jose, California, USA), of which three detection bandpass (BP) filters were used for data acquisition (BP 586/15 for PE, BP 780/60 for PE/Cy7, and BP 670/30 for APC detection; BP530/30 was used to exclude high autofluorescence events/debris). Antibodies for basophil staining were: PE anti-human CD203c (clone NP4D6), PE/Cy7 anti-human FcεRIα (clone AER-37), and APC anti-human CD63 (clone H5C6), all antibodies from BioLegend (Fell, Germany). The BAT and the identification of basophils (gating strategy) was performed as previously published by us^19^, and described within the supplemental Figure S1. Basophils were not only identified as described before^19^, but additionally their activation status was analyzed via upregulation of CD63 and CD203c expression, respectively. Data analysis was conducted with the FCS Express V7.22.0006.

### Statistical analysis

Liposome assays were performed as *n* = 2 (Fig. 1) and *n* = 3 (Figs. 2 and 3) biologically independent experiments. Data were analyzed for statistical significance with GraphPad Prism Version 10.4.2. Statistical tests are indicated in the figure legends, statistical significance is indicated as *p ≤ 0.05, **p ≤ 0.01; ***p ≤ 0.001; **** *p* ≤ 0.0001; ns, not significant. As *L. luteus* nLTP is not available for routine allergy diagnostics yet, patients identified with this phenotype are still rare. The BAT assay was performed as a case study with blood of one representative patient. The data could, therefore, not be analyzed for statistical significance.

## Supplementary Information

Below is the link to the electronic supplementary material.

Supplementary Material 1

## Data Availability

All data generated or analyzed during this study are included in this published article. Data can be requested by the authors at reasonable request.

## References

[1] Dramburg, S. et al. EAACI Molecular Allergology User’s Guide 2.0. *Pediatr. Allergy Immunol.* **34**(Suppl 28), e13854 (2023). 37186333 10.1111/pai.13854

[2] Pastorello, E. A. et al. The major allergen of peach (Prunus persica) is a lipid transfer protein. *J. Allergy Clin. Immunol.* **103**(3 Pt 1), 520–526 (1999). 10069889 10.1016/s0091-6749(99)70480-x

[3] Gülsen, A. & Jappe, U. Lipid transfer protein sensitization in an apple-allergic patient: a case report from northern Europe. *Eur. Ann. Allergy Clin. Immunol.* **51**(2), 80–83 (2019). 29542892 10.23822/EurAnnACI.1764-1489.63

[4] Albert, E. et al. Lipid transfer protein syndrome in a Northern European patient: An unusual case report. *Front. Med. (Lausanne)*. **10**, 1049477 (2023). 36824608 10.3389/fmed.2023.1049477 PMC9941155

[5] Scheurer, S., van Ree, R. & Vieths, S. The Role of Lipid Transfer Proteins as Food and Pollen Allergens Outside the Mediterranean Area. *Curr. Allergy Asthma Rep.* **21**(2), 7 (2021). 33537877 10.1007/s11882-020-00982-w PMC7858557

[6] Skypala, I. J. et al. Non-specific lipid-transfer proteins: Allergen structure and function, cross-reactivity, sensitization, and epidemiology. *Clin. Transl Allergy*. **11**(3), e12010 (2021). 34025983 10.1002/clt2.12010 PMC8129635

[7] KaderJ.C. Lipid-transfer proteins in plants. *Annu. Rev. Plant. Physiol. Plant. Mol. Biol.* **47**, 627–654 (1996). 10.1146/annurev.arplant.47.1.627 15012303

[8] Scheurer, S. & Schülke, S. Interaction of Non-Specific Lipid-Transfer Proteins With Plant-Derived Lipids and Its Impact on Allergic Sensitization. *Front. Immunol.* **9**, 1389 (2018). 29973934 10.3389/fimmu.2018.01389 PMC6019453

[9] Fahy, E. et al. A comprehensive classification system for lipids. *J. Lipid Res.* **46**(5), 839–861 (2005). 15722563 10.1194/jlr.E400004-JLR200

[10] Liu, F. et al. Non-specific lipid transfer proteins in plants: presenting new advances and an integrated functional analysis. *J. Exp. Bot.* **66**(19), 5663–5681 (2015). 26139823 10.1093/jxb/erv313

[11] Jappe, U. et al. Lipophilic Allergens, Different Modes of Allergen-Lipid Interaction and Their Impact on Asthma and Allergy. *Front. Immunol.* **10**, 122 (2019). 30837983 10.3389/fimmu.2019.00122 PMC6382701

[12] Dubiela, P. et al. Enhanced Pru p 3 IgE-binding activity by selective free fatty acid-interaction. *J. Allergy Clin. Immunol.* **140**(6), 1728–1731e10 (2017). 28712832 10.1016/j.jaci.2017.06.016

[13] Dubiela, P. et al. Impact of lipid binding on the tertiary structure and allergenic potential of Jug r 3, the non-specific lipid transfer protein from walnut. *Sci. Rep.* **9**(1), p2007 (2019). 10.1038/s41598-019-38563-1 PMC6376136 30765752

[14] Finkina, E. I. et al. *Impact of Different Lipid Ligands on the Stability and IgE-Binding Capacity of the Lentil Allergen Len c 3*. *Biomolecules*, **10**(12). (2020). 10.3390/biom10121668 PMC7763088 33322094

[15] Martín-Pedraza, L. et al. Two nonspecific lipid transfer proteins (nsLTPs) from tomato seeds are associated to severe symptoms of tomato-allergic patients. *Mol. Nutr. Food Res.* **60**(5), 1172–1182 (2016). 26840232 10.1002/mnfr.201500782

[16] Parrón-Ballesteros, J. et al. Long-chain fatty acids block allergic reaction against lipid transfer protein Sola l 7 from tomato seeds. *Protein Sci.* **33**(9), e5154 (2024). 39180496 10.1002/pro.5154 PMC11344279

[17] Poncet, P., Sénéchal, H. & Charpin, D. Update on pollen-food allergy syndrome. *Expert Rev. Clin. Immunol.* **16**(6), 561–578 (2020). 32691654 10.1080/1744666X.2020.1774366

[18] Schromm, A. B. et al. Cathelicidin and PMB neutralize endotoxins by multifactorial mechanisms including LPS interaction and targeting of host cell membranes. Proc. Natl. Acad. Sci. U S A, **118**(27). (2021). 10.1073/pnas.2101721118 PMC8271772 34183393

[19] Behrends, J. et al. Innovative robust basophil activation test using a novel gating strategy reliably diagnosing allergy with full automation. *Allergy* **76**(12), 3776–3788 (2021). 33973252 10.1111/all.14900

[20] Jappe, U. Vegan diet—alternative protein sources as potential allergy risk. *Allergo J. Int.* **32**(7), 251–257 (2023).

[21] Jappe, U. & Vieths, S. Lupine, a source of new as well as hidden food allergens. *Mol. Nutr. Food Res.* **54**(1), 113–126 (2010). 20013885 10.1002/mnfr.200900365

[22] Petersen, A. et al. Roasting and lipid binding provide allergenic and proteolytic stability to the peanut allergen Ara h 8. *Biol. Chem.* **395**(2), 239–250 (2014). 24057594 10.1515/hsz-2013-0206

[23] Bublin, M., Eiwegger, T. & Breiteneder, H. Do lipids influence the allergic sensitization process? *J. Allergy Clin. Immunol.* **134**(3), 521–529 (2014). 24880633 10.1016/j.jaci.2014.04.015 PMC4151997

[24] Aina, R. et al. Distinct Lipid Transfer Proteins display different IgE-binding activities that are affected by fatty acid binding. *Allergy* **74**(4), 827–831 (2019). 30480827 10.1111/all.13682 PMC6491988

[25] Calvano, C. D. et al. Analysis of Phospholipids, Lysophospholipids, and Their Linked Fatty Acyl Chains in Yellow Lupin Seeds (*Lupinus luteus L*.) by Liquid Chromatography and Tandem Mass Spectrometry. Molecules, **25**(4). (2020). 10.3390/molecules25040805 PMC7070507 32069835

[26] Kupsch, S. et al. Characterization of phospholipid-modified lung surfactant in vitro and in a neonatal ARDS model reveals anti-inflammatory potential and surfactant lipidome signatures. *Eur. J. Pharm. Sci.* **175**, 106216 (2022). 35618202 10.1016/j.ejps.2022.106216

[27] Roughan, P. G. Phosphatidylglycerol and chilling sensitivity in plants. *Plant. Physiol.* **77**(3), 740–746 (1985). 16664127 10.1104/pp.77.3.740 PMC1064594

[28] Yu, B. & Benning, C. Anionic lipids are required for chloroplast structure and function in Arabidopsis. *Plant. J.* **36**(6), 762–770 (2003). 14675442 10.1046/j.1365-313x.2003.01918.x

[29] Kobayashi, K. et al. Specific role of phosphatidylglycerol and functional overlaps with other thylakoid lipids in Arabidopsis chloroplast biogenesis. *Plant. Cell. Rep.* **34**(4), 631–642 (2015). 25477206 10.1007/s00299-014-1719-z

[30] Wang, K. et al. A Plastid Phosphatidylglycerol Lipase Contributes to the Export of Acyl Groups from Plastids for Seed Oil Biosynthesis. *Plant. Cell.* **29**(7), 1678–1696 (2017). 28687655 10.1105/tpc.17.00397 PMC5559756

[31] Dugail, I., Kayser, B. D. & Lhomme, M. Specific roles of phosphatidylglycerols in hosts and microbes. *Biochimie* **141**, 47–53 (2017). 28483688 10.1016/j.biochi.2017.05.005

[32] Kayser, B. D. et al. Phosphatidylglycerols are induced by gut dysbiosis and inflammation, and favorably modulate adipose tissue remodeling in obesity. *Faseb J.* **33**(4), 4741–4754 (2019). 30608881 10.1096/fj.201801897R PMC8793811

[33] Eggers, L. F. et al. Lipidomes of lung cancer and tumour-free lung tissues reveal distinct molecular signatures for cancer differentiation, age, inflammation, and pulmonary emphysema. *Sci. Rep.* **7**(1), 11087 (2017). 28894173 10.1038/s41598-017-11339-1 PMC5594029

[34] Numata, M., Kandasamy, P. & Voelker, D. R. The anti-inflammatory and antiviral properties of anionic pulmonary surfactant phospholipids. *Immunol. Rev.* **317**(1), 166–186 (2023). 37144896 10.1111/imr.13207 PMC10524216

[35] Wawrzeńczyk, A. et al. Sensitisation to lipid transfer proteins in pollen - allergic adults with food allergy. *Postepy Dermatol. Alergol*. **37**(4), 508–512 (2020). 32994771 10.5114/ada.2020.98278 PMC7507168

[36] Crespo, J. F. et al. Occupational IgE-mediated allergy after exposure to lupine seed flour. *J. Allergy Clin. Immunol.* **108**(2), 295–297 (2001). 11496250 10.1067/mai.2001.116860

[37] Schumann, R. R. et al. Structure and function of lipopolysaccharide binding protein. *Science* **249**(4975), 1429–1431 (1990). 2402637 10.1126/science.2402637

[38] Yu, B., Hailman, E. & Wright, S. D. Lipopolysaccharide binding protein and soluble CD14 catalyze exchange of phospholipids. *J. Clin. Invest.* **99**(2), 315–324 (1997). 9006000 10.1172/JCI119160 PMC507799

[39] Schromm, A. B. et al. Lipopolysaccharide-binding protein mediates CD14-independent intercalation of lipopolysaccharide into phospholipid membranes. *FEBS Lett.* **399**(3), 267–271 (1996). 8985160 10.1016/s0014-5793(96)01338-5

[40] Mueller, M. et al. Phospholipids inhibit lipopolysaccharide (LPS)-induced cell activation: a role for LPS-binding protein. *J. Immunol.* **174**(2), 1091–1096 (2005). 15634934 10.4049/jimmunol.174.2.1091

[41] Spengler, D. et al. Novel therapeutic roles for surfactant-inositols and -phosphatidylglycerols in a neonatal piglet ARDS model: a translational study. *Am. J. Physiol. Lung Cell. Mol. Physiol.* **314**(1), L32–l53 (2018). 28860142 10.1152/ajplung.00128.2017

[42] Jappe, U. et al. Identification and Purification of Novel Low-Molecular-Weight Lupine Allergens as Components for Personalized Diagnostics. Nutrients, **13**(2). (2021). 10.3390/nu13020409 PMC7911308 33525401

