# Supplementary material for: Lipid ligand binding and membrane interactions of a novel food-derived lipid transfer protein enhance basophil allergic responses
Source: Sci Rep. 2026 Jun 13;16:18352. doi: 10.1038/s41598-026-55182-9 (PMC13264632; doi:10.1038/s41598-026-55182-9)
Supplement: Supplementary file 1 — Supplementary Material 1 [file 41598_2026_55182_MOESM1_ESM.pptx]

## Slide 1
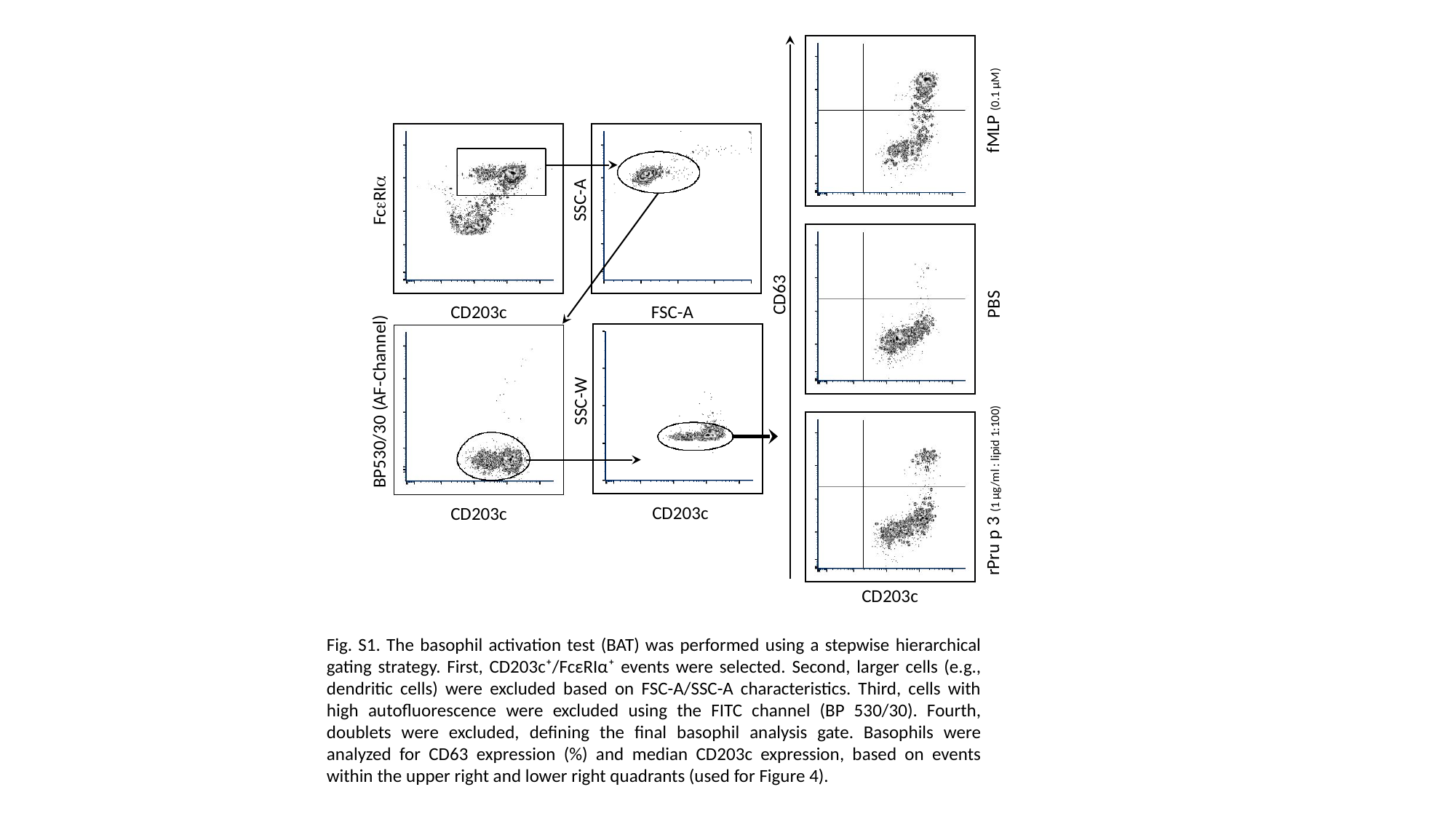

fMLP (0.1 µM)
FceRIa
SSC-A
CD63
PBS
CD203c
FSC-A
SSC-W
BP530/30 (AF-Channel)
rPru p 3 (1 µg/ml : lipid 1:100)
CD203c
CD203c
CD203c
Fig. S1. The basophil activation test (BAT) was performed using a stepwise hierarchical gating strategy. First, CD203c⁺/FcεRIα⁺ events were selected. Second, larger cells (e.g., dendritic cells) were excluded based on FSC-A/SSC-A characteristics. Third, cells with high autofluorescence were excluded using the FITC channel (BP 530/30). Fourth, doublets were excluded, defining the final basophil analysis gate. Basophils were analyzed for CD63 expression (%) and median CD203c expression, based on events within the upper right and lower right quadrants (used for Figure 4).
